# Unusual Cause of Persistent Epistaxis with Severe Anemia in a Child

**DOI:** 10.1155/2022/8557755

**Published:** 2022-08-08

**Authors:** Yilkal Zemene, Tadele Hailu, Josh Wiedermann

**Affiliations:** ^1^ENT and Head and Neck Surgeon, St Paul's Hospital Millennium Medical College, Addis Ababa, Ethiopia; ^2^Pediatric Hemato Oncologist, St. Paul's Hospital Millenium Medical College, Addis Ababa, Ethiopia; ^3^Otolaryngology-Head and Neck Surgery, Mayo Clinic, Rochester, Minnesota, USA

## Abstract

Epistaxis in children can be caused by different systemic and local pathologies. Respiratory infections, nasal mucosa dryness, and foreign bodies are some local causes of bleeding from the nose. In developing countries, infestations still contribute a significant proportion of anemia in children. But it is very unusual for leech-causing persistent epistaxis with a consequence of severe anemia. We herein report a rare cause of severe anemia in a 5-year-old child presented to our clinic for persistent epistaxis. A leech was taken out with forceps, and his anemia was treated accordingly.

## 1. Introduction

Anemia is a public health problem in developing countries. Worldwide two billion people are estimated to suffer from anemia. Common causes of anemia in developing countries include nutritional deficiency, hemoglobin disorders, and infectious problems including parasitic infestations [1]. Leech infestation is one of the parasitic infestation people encounter in low socioeconomic countries where access to safe water is limited. The symptoms of presentation depend on the site of infestation: the usual ones being hemoptysis, upper airway obstruction, and vaginal bleeding [2–4].

## 2. Case Presentation

**Figure 1:**
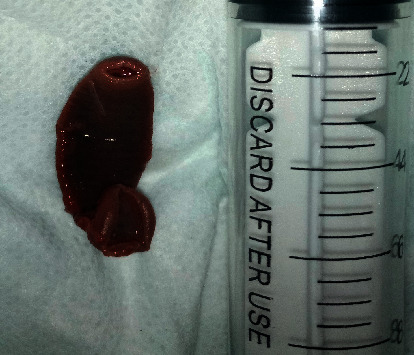
The leech removed from the nasopharyngeal area of our 5-year-old patient. Note: The head sucker and the posterior sucker are clearly visible. And the length can be estimated from the 10 cc syringe kept beside it.

A 5-year-old child was presented to the emergency department of Ayder specialized referral teaching hospital in Mekelle, Ethiopia, for a complaint of nasal bleeding for one week. He had loss of appetite, easy fatigability, change in stool color, and mild cough with blood mixed saliva. On physical examination, he was tachycardic (152 per minute) but was not in any form of distress. He had pale conjunctiva and oral mucosa. Otherwise, there was no any other pertinent physical finding. The first impression of the physician at the emergency unit was hematologic malignancies and hookworm infestation. Complete blood count was done. Hemoglobulin was 3 gm/dl (NL = 11.5–14.5 gm/dl), WBC 11300/ul (NL = 55000–15,500/ul), platelets 279,000/mm^3^ (NL = 187–445,000/mm^3^), MCH 28.2 pg (NL = 27–31 pg), MCV 82.5 FL (NL = 77.2–89.5 FL), and MCHC 34.2 gm/dl (NL = 32–36 gm/dl). Stool tested negative for hook warm. For the persistent epistaxis, the ENT department was consulted. During physical examination, it was observed that the epistaxis was not profuse and it was atypical of the bleeding nose we usually encounter. Oropharyngeal examination showed bloodstained oral and oropharyngeal mucosa. Part of a dark greenish live wiggling leech at the upper part of the oropharynx was observed, and it was taken out with forceps safely and there was no bleeding thereafter. The leech measured 4 cm (Figure 1). With further detailed history taking, we found that the local pond was used for drinking water. As to the severe anemia, he was admitted to the ward, transfused with whole blood, and discharged with hem-up syrup after one week, and the parents are advised how to make the local stagnant water source safe for drinking and bathing.

## 3. Discussion

Anemia is defined as 2 standard deviations less than the normal for age and gender. It is one of the commonest public health problems across the globe. Worldwide the prevalence of anemia in children under 5 years of age is estimated to be 42.6% [5]. In Ethiopia, the overall prevalence was 51.5% significantly different across age segregation [6].

General causes of Anemia in children can be due to decreased red blood cell production, destruction of redblood cells, or loss of red blood cells. Iron deficiency is the single most common nutritional disorder worldwide and is the main cause of anemia across all the age groups [7]. Leech infestation causes anemia due to continous and significant bleedingas well asitsavid for iron consumption.

Leech, Annelida hirudinea, is a hermaphrodite hemophagus worm which affects fish, amphibians, and mammals. It is a carnivorous for small invertebrates but sucks blood in bigger animals like mammals [8]. The medicinal leech (*Hirudo medicinalis*)) has two suckers, one at each end, called the anterior and posterior suckers. The posterior sucker is mainly used for leverage while the anterior sucker, consisting of the jaw and teeth, is used for feeding. They use mucus and suction pressure (produced by concentric muscles in the first six segments) to stay attached and secrete hirudin and other enzymes, which have anti-thrombin activity in the host's blood stream. It also releases an anesthetic to prevent the host from feeling pain while it sucks the blood [9].

Because a leech is only interested in hemoglobulin, they ingest the whole blood, filter out, and excrete the plasma which makes the hematocrit in the crub diverticulum up to 100%, and its feeding is assisted by pharyngeal and body wall peristalsis which pumps the blood into the diverticulum. Enzymes and the dorsiflexion of the leech will prevent the stored blood from clotting [8].

The clinical presentation depends on the site of infestation. Demeke reported acute airway obstruction in a child, and the leech was removed with forceps [3], while Sarathi reported dysphagia and epistaxis [10]. Nebiyu and Mustafa reported severe anemia in a child who needs transfusion [2, 11]. Hanna et al. shared their experience in managing children with leech infestation involving urethra, vagina, or rectum [12].

Our patient had melena which shows how significant the bleeding was, and the epistaxis during leech manifestation was small but persistent one. The patient has saliva mixed with blood rather than the usual clotted blood coming out from the mouth.

Mechanisms of leech removal from the oropharynx include local anesthetic agents like xylocaine, lidocaine, cocaine [2], and electrocautery [13]. The people in the rural Ethiopia use lemon and salty water to detach it from its attachment, and they usually visit the clinics when their trials fail. The removal method we prefer to use in our setup is to hold the leech with artery forceps for few seconds, and it falls off the forceps by itself and can be removed safely. Otherwise, the mouth with the jaw may remain in the site of attachment, causing continuous bleeding from the site.

In poor tropical and subtropical areas, where people walk by barefoot, it is not uncommon to get people with massive hookworm infestations. But, other than the occasional severe anemia, frank bleeding is not its common manifestation and stool tested negative for hookworm [14].

Considering the geographical location from where the patient comes from, we were encountering malaria which is a major cause of anemia in his village, especially during the rainy season. It causes hemolysis of infected and uninfected erythrocytes [15]. But we did not send samples for blood film examination as the treating emergency team wanted to rule out local causes for the epistaxis first. He also did not have fever and other common symptoms and signs of malaria.

Hemolytic anemia is another common cause of anemia. It can be caused by intrinsic hemoglobinopathies or extrinsic factors like drugs [16]. But bleeding is uncommon.

Even though epistaxis is not a common feature of upper gastrointestinal bleeding (UGIB), we considered it as he had severe anemia and melena. A study done by Yachha et al. showed 95% of UGIB in developing countries has varices while the remaining children has different causes like Henoch-Schoenlein purpura, immune thrombocytopenic purpura, and drug-induced gastric erosion [17]. We did not have any pertinent finding during abdominal examination.

Epistaxis and anemia are common manifestations of patients with bleeding disorders. In children with excessive bleeding, health professionals have to suspect bleeding disorders based on the history, physical examination, and laboratory findings. Acute blood loss anemia can be caused by liver failure [18], but he was apparently healthy previously and did not have any of the manifestations of fulminant hepatitis either. Mild hemophilia can present late, but our patient had no history of previous bleeding, easy bruising, and bleeding in the joints [19]. Prothrombin time, activated partial thromboplastin time (aPTT), and platelet concentration are the first line of laboratory investigation for such patients. If the investigation has abnormal results, then the child should be evaluated further by a hematologist [20]. But our patient was not investigated on this line as it was not available in the emergency laboratory of our hospital.

## 4. Conclusion

Leech manifestation is not uncommon in the rural areas where access to safe water is very much limited, and anybody with mild but persistent epistaxis, blood mixed saliva, and severe anemia has to be suspected for leech infestation. We also recommend that health education about safe water should be strengthened by the local healthcare providers.

## Data Availability

All data are included within the article.
